# C1q/TNF-Related Protein-3 (CTRP-3) and Pigment Epithelium-Derived Factor (PEDF) Concentrations in Patients with Gestational Diabetes Mellitus: A Case-Control Study

**DOI:** 10.3390/jcm9082587

**Published:** 2020-08-10

**Authors:** Tomasz Gęca, Maciej Kwiatek, Arkadiusz Krzyżanowski, Anna Kwaśniewska

**Affiliations:** Chair and Department of Obstetrics and Pathology of Pregnancy, Medical University of Lublin, Staszica 16 Street, 20-081 Lublin, Poland; kwiatula1@wp.pl (M.K.); a_r_krzyzanowski@tlen.pl (A.K.); haniakwasniewska@gmail.com (A.K.)

**Keywords:** gestational diabetes mellitus, pregnancy, PEDF, CTRP-3

## Abstract

Background: Gestational diabetes mellitus (GDM) is the most common metabolic disorder in pregnant women, defined as any degree of glucose intolerance with onset or first detected during pregnancy. Explanation of its pathogenesis is extremely important due to the possibility of preventing serious maternal and fetal complications. The aim of the study was to evaluate the concentrations of two molecules: C1q/tumor necrosis factor-related protein-3 (CTRP-3) and pigment epithelium-derived factor (PEDF) which may possibly participate in GDM development. To our knowledge, this is the first study in pregnant women with GDM evaluating CTRP-3 level. Methods: Serum CTRP-3 and PEDF concentration and clinical characteristics were detected in 172 pregnant women. These women were divided into two groups: normal glucose tolerance group (NGT, *n* = 54) and gestational diabetes mellitus group (GDM, *n* = 118). This second group was further divided into two subgroups depending on the treatment used: GDM 1—diet only (*n* = 75) and GDM 2—insulin treatment (*n* = 43). Results: Our study did not reveal any statistically significant difference between the concentration of PEDF in the control and GDM group. In our study there was a significantly higher concentration of CTRP-3 evaluated in the peripheral blood serum in patients with gestational diabetes (GDM) compared to those in the control group (8.84 vs. 4.79 ng/mL). Significantly higher values of CTRP-3 were observed in both the diet-treated subgroup and the group with insulin therapy when compared to control group (8.40 and 10.96, respectively vs. 4.79 ng/mL). Conclusion: PEDF concentration does not change in GDM, whereas an increased level of CTRP-3 may point to the key role of this adipokine in the development of GDM.

## 1. Introduction

Gestational diabetes mellitus (GDM) is a special form of diabetes in pregnant women defined as any degree of glucose intolerance with onset or first recognition during pregnancy [1]. GDM affects up to 15% of pregnant women worldwide [2]. It is a particularly important public health issue that is associated with serious consequences for both mother (gestational hypertension, preeclampsia, delivery trauma) and offspring (macrosomia, preterm birth, shoulder dystocia, congenital malformations) [3,4,5]. GDM is also associated with long-term consequences such as metabolic syndrome, cardiovascular disease and type 2 diabetes in the offspring [6]. Most women with GDM revert to normal glucose metabolism during puerperium; however, they are at higher risk of developing type 2 diabetes later in life [7]. 

The precise mechanisms underlying this form of diabetes in pregnancy remain unclear, but pancreatic β-cell insufficiency in compensating for pregnancy-induced insulin resistance is considered to be important [8]. Explanation of GDM pathogenesis is important due to the possibility of preventing maternal and fetal complications. Two molecules, C1q/tumor necrosis factor-related protein-3 (CTRP-3) and pigment epithelium-derived factor (PEDF), may possibly participate in GDM development due to the fact that underlying mechanisms of GDM are, in general, similar to the mechanisms responsible for metabolic disorders such as type 2 diabetes mellitus or obesity. 

The complement C1q tumor necrosis factor related protein (CTRP) superfamily is a newly found cluster of adipokines with a common structure composed of collagenous and globular C1q-like domains. CTRP-3 was first discovered in 2001 in C3H10T1/2 mouse mesenchymal stem cells treated to induce chondrogenic differentiation and was originally named CORS26 (Collagenous repeat-containing sequence 26 kDa protein) due to its specific structure—23 Gly-X-Y repeats in the N-terminal collagen domain [9]. After Wong et al. identified CORS26 as a member of CTRP family with highly conserved adiponectin paralogs it was renamed CTRP-3. N-terminal Collagenous repeats (Gly-X-Y), and a highly conserved C-terminal globular domain, place CTRP3 within the C1q TNF Superfamily [10]. It shares sequence homology with adiponectin and is highly conserved with almost 96% identity between human and mouse proteins [11]. In addition, two splice variants of CTRP-3 were identified: CTRP-3A and CTRP-3B. Unlike CTRP-3A, CTRP-3B contains a highly conserved N-linked glycosylation site. CTRP-3B is the longer splice variant and encodes an extra 73 N-terminal amino acids due to the retention of intron 1. Both splice variants of CTRP-3 are secreted proteins, but their functional significance remains unknown [12]. Recent studies have suggested that this paralog of adiponectin may play an important role in the regulation of glucose metabolism and thus in GDM pathogenesis [12]. This novel adipokine is characterized by multiple metabolic effects such as lowering glucose levels, inhibiting gluco-neogenesis and increasing angiogenesis and anti-inflammation [12,13]. It is also known as a cartonectin, and cartducin, both due to the detection of CTRP-3 expression in developing cartilage [14].

Pigment epithelium-derived factor (PEDF), a multifunctional protein, consisting of 418 amino acids, is associated with insulin resistance and metabolic syndrome. PEDF induces insulin resistance in human adipocytes and skeletal muscle cells [15,16]. The combination of increased insulin resistance and insufficient insulin response during pregnancy seems to be the main pathophysiological mechanism responsible for GDM development. PEDF is a 50 kDa secreted glycoprotein that belongs to the non-inhibitory serpin group [17]. The gene encoding PEDF, *Serpinf1*, is located on chromosome 17p13 and is widely expressed in many tissues [18]. The highest expression levels in humans are found in the liver and adipose tissue [19]. It was first purified from a conditioned media of retinal epithelial cells and identified as a neurotrophic factor with potent neuronal differentiative activity (ability to convert retinoblastoma tumor cells into differentiated non-proliferative neurons) [20]. It is assumed that PEDF functions via receptors: ATGL (adipose triglyceride lipase) and laminin-R. Patatin-like phospholipase domain containing protein-2 (PNPLA2), also known as ATGL, was first identified in 2006 by Notari et al. [21]. The second known receptor of PEDF, laminin-R, was described three years later and is associated with a number of processes such as cell proliferation, differentiation, adhesion and migration [22]. The multiplicity of PEDF functions led the researchers to assume that there were more than two receptors for PEDF and several putative receptors were suggested as taking part in the PEDF acting mechanism that include lipoprotein receptor-related protein 5 (LPR5) and cell surface F1F0-ATP synthase, which are involved in PEDF anti-angiogenic activity [23]. The regulatory effect of PEDF on lipid and carbohydrate metabolism is mediated by ATGL which activates adipose lipolysis. This mechanism may contribute to insulin resistance in obese subjects [24]. Borg et al. noted that ATGL-deficient mice do not develop PEDF-induced insulin resistance [24]. 

However, despite such a large role that these two proteins play in carbohydrate metabolism, no previous reports have measured circulating CTRP-3 levels in GDM and only one evaluates PEDF as a potential early detection marker for predicting development of GDM to diabetes mellitus [25]. We have therefore decided to measure CTRP-3 and PEDF in pregnant women with GDM in comparison to normoglycemic women, and find correlations. 

## 2. Experimental Section

All procedures performed in studies involving human participants were in accordance with the ethical standards of the institutional and/or national research committee (Ethics Committee of Medical University of Lublin in Lublin. Poland) and with the 1964 Helsinki declaration and its later amendments or comparable ethical standards. Ethical Approval Code is 0254/120/2016.

This article does not contain any studies with animals performed by any of the authors.

**Figure 1:**
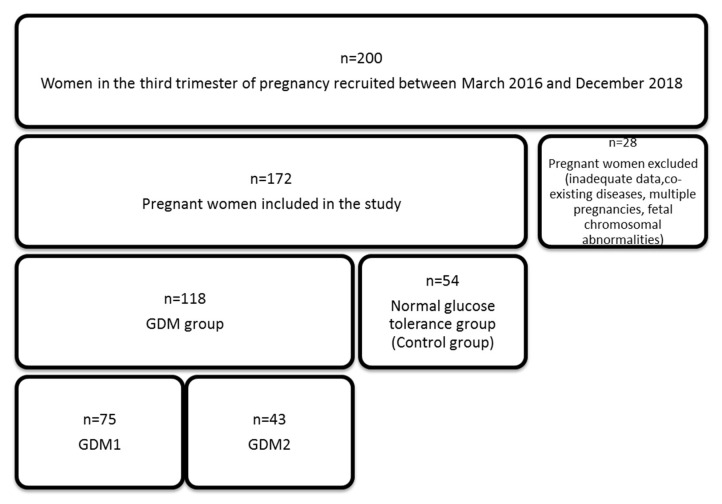
Flowchart of participant recruitment and case–control study.

A total of 200 women in the third trimester of pregnancy were recruited between March 2016 and December 2018 from the Department of Obstetrics and Pathology of Pregnancy of the Independent Public Teaching Hospital No 1 in Lublin. Of the 200 pregnancies, 28 were excluded because of inadequate data (no BMI counted before pregnancy, incomplete oral glucose tolerance test 75 g results), co-existing diseases (pregestational diabetes, hypertension, preeclampsia, thyroid gland diseases, chronic renal disease, and collagenosis), multiple pregnancies, or fetal chromosomal abnormalities. In total, 172 pregnant women were included in the study. These women were divided into two groups: normal glucose tolerance group (NGT, *n* = 54) and gestational diabetes mellitus group (GDM, *n* = 118). This second group was further divided into two subgroups depending on the treatment used: GDM 1—diet only (*n* = 75) and GDM 2—insulin treatment (*n* = 43). The diagnosis of GDM was based on the WHO criteria [1]. The present study was conducted in accordance with the Declaration of Helsinki of the World Medical Association and was approved by the Ethics Committee of the Medical University of Lublin in Lublin, Poland (Nr 0254/120/2016). A written informed consent was obtained from all participants. The basic workflow of this study is presented in Figure 1.

The database of clinical background characteristics included gravidity, parity, maternal age, pre-pregnancy body mass index (BMI), gestational weight gain, OGTT 75 g results, gestational age at delivery, delivery mode including vaginal delivery or cesarean section, and newborn parameters including sex, birth weight, and APGAR score (1 and 5 min after birth). Pre-pregnancy body weight was determined based on self-reporting at the first obstetrical visit. Gestational age was determined based on the last menstrual period or the measurement of crown-rump length assessed by ultrasound in early pregnancy in cases of unknown date of last menstrual period or irregular menstrual period. The women with GDM received guidance regarding self-monitoring of blood glucose levels four to six times a day from a licensed nurse. Dietary counseling was provided for each woman with GDM. Height and body weight were measured by standardized methods in all subjects. The body mass index (BMI) formula was computed as weight in kilograms divided by height in meters squared. The homeostasis model assessment of insulin resistance (HOMA-IR) = fasting insulin (mU/L) * fasting plasma glucose (mmol/L)/22.5.

Blood samples were collected in the morning (7–9 a.m.) after an overnight fasting (≥8 h). Plasma samples were obtained by centrifugation at 2000× *g* for 10 min at 4 °C and were kept at −80 °C before analyses. Plasma glucose levels were measured using the spectrophotometric method (Glucose Assay Kit (Cat No. SUP6016, Empire Genomics with the use of Epoch Microplate Spectrophotometer, BioTek Instruments (Winooski, VT, USA), fasting insulin was measured using Insulin ELISA (Cat No. EIA-2935, DRG Instruments GmbH, Marburg, Germany). The concentration of Human pigment epithelium-derived factor (PEDF) in samples was determined using ELISA kits purchased from Sunred Biological Technology Co., Ltd. (Cat. No. 201-12-1635, Shanghai, China). Concentrations of human CTRP3 were measured with a commercial ELISA kit (Aviscera Bioscience, Cat. No.SK00082-07, Santa Clara, CA, USA). The assays were performed according to the manufacturer’s instructions.

### Statistical Methods

The statistical analysis was performed using statistical software (Statistica 13). The correlations of selected biomarkers and continuous clinical variables (measured by means of Spearman non-parametric test or Pearson parametric test depending on the distribution of data) were analysed within all the subgroups. Particular groups were assessed on the basis of the distribution of the above-mentioned variables (Shapiro-Wilk test). Since all tested variables, in at least one of the compared subgroup, showed non-normal distribution, the results were presented as medians. In order to compare the data distribution of continuous variables with a non-normal distribution, a non-parametric U-Mann Whitney (comparison of 2 groups) or ANOVA Kruskal-Wallis (comparison of more than 2 groups) tests was used. The distribution of variables categorized in relevant subgroups was compared by means of *Chi* square test. *P* values < 0.05 were considered statistically significant.

## 3. Results

### 3.1. The Characteristics and Comparison of Selected Clinical and Laboratory Variables Depending on the Occurrence of Diabetes during Pregnancy and the Implemented Treatment (A Diet or an Insulin Therapy)

**Figure 2:**
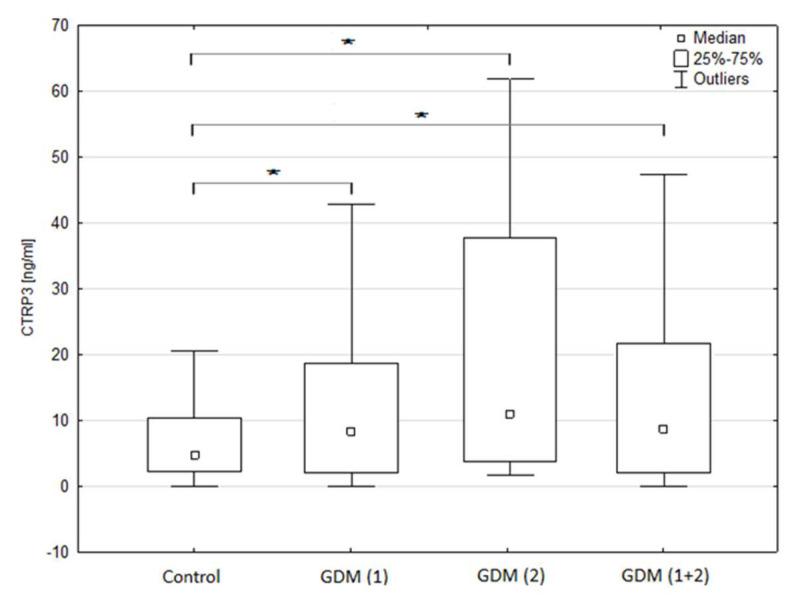
Comparisons of C1q/tumor necrosis factor-related protein-3 (CTRP-3) serum concentration according to occurrence of gestational diabetes mellitus (GDM) and treatment. *—statistically significant differences (*p* < 0.05).

The median age of the control group was 29 years. The median age in the GDM group was 32 years, of which GDM1 and GDM2 were 31 and 32 years, respectively. The study group did not differ in terms of basic factors such as: age, number of pregnancies, number of labours, type of delivery, child’s sex, weight of a new-born, height and weight of mother (both before pregnancy and currently). The groups included in the study did not display any significant differences as to PEDF and insulin concentrations. However, in the case of BMI (assessed before pregnancy), higher values (>24.99: overweight) were significantly more often observed in women with GDM, regardless of the treatment (Control (11.11%) vs. GDM1 (34.67%, *p* = 0.0055), GDM2 (44.19%, *p* = 0.0005) and GDM1 + 2 (38.14%, *p* = 0.0014)). In the GDM group compared to the control group, median BMI (assessed before pregnancy) was significantly higher (23.44 vs. 23.28; *p* = 0.0341). Interestingly, median BMI was also significantly higher in GDM2 subgroup compared to both GDM1 and control (24.22 vs. 23.23 and 23.28, respectively; *p* = 0.0164). A substantially higher concentration of CTRP3 in peripheral blood plasma was noted in the patients with diagnosed gestational diabetes mellitus (GDM) as compared to the respondents from the control group (8.84 vs. 4.79 ng/mL; *p* = 0.0265; Figure 2). Considerably higher values of this marker (compared to the control group) were observed in both the diet-treated subgroup (GDM 1: 8.40 vs. 4.79 ng/mL) and the insulin therapy group (GDM 2: 10.96 vs. 4.79 ng/mL) (*p* = 0.0178). Detailed data including the differences between the study groups in terms of selected clinical and laboratory parameters are presented in Table 1 and Table 2.

### 3.2. Correlations between the Selected Clinical and Laboratory Variables as Well as PEDF Marker and CTRP3 in the Groups of: Healthy Respondents, Patients with Gestational Diabetes Mellitus (GDM), Gestational Diabetes Mellitus Treated by Means of Diet (GDM1) and by Means of Insulin Therapy (GDM2)

A weak, negative correlation between PEDF concentration and body weight (initial weight) was noted in GDM group (rho = −0.229, *p* = 0.0438). On the other hand PEDF concentration correlated strongly and positively with APGAR 2 (rho = 0.825, *p* = 0.0117). In fact, this correlation refer to GDM2 group because APGAR 2 data was not available for the GDM1 group. Moreover, PEDF concentration was also weakly and negatively correlated with BMI indicator (assessed before pregnancy) in GDM2 group (rho = −0.351, *p* = 0.0210). In the control group moderate, negative correlation between PEDF concentration and age was found (rho = −0.412, *p* = 0.0020). Interestingly only in the case of healthy women (control group), a trend to the statistically significant result (weak positive correlation) between PEDF concentration and the increase of weight during pregnancy was noted (rho = 0.290, *p* = 0.0504). Both in GDM and GDM2 groups there was weak, positive correlation between CTRP3 concentration and insulin (rho = −0.208, *p* = 0.0243; rho = 0.333, *p* = 0.0293, respectively). However, in both above mentioned groups we found weak, negative correlation between CTRP3 concentration and gestational age (rho = −0.254, *p* = 0.0070; rho = −0.382, *p* = 0.0126, respectively). On the other hand in GDM1 group weak, negative correlation between CTRP3 concentration and current weight (rho = −0.256, *p* = 0.0341) as well as increase of weight during pregnancy (rho = −0.352, *p* = 0.0326) was found. Interestingly, in none of the studied groups, CTRP3 did not correlate with baseline weight or BMI. Detailed data referring to the correlations between the selected clinical and laboratory variables as well as PEDF markers and CTRP3 in the groups of healthy respondents, patients with gestational diabetes mellitus (GDM 1 + 2), gestational diabetes mellitus treated by means of diet (GDM1) and by means of insulin therapy (GDM2) are presented in Table 3 and Table 4. PEDF concentration does not change in GDM but it tends to grow predominantly in the case of long-term diabetes and its complications. In addition, the increased level of CTRP3 in GDM may be indicative of this component’s role in the development of GDM.

## 4. Discussion

GDM is a condition of carbohydrate intolerance with onset or first recognition in pregnancy and the most common metabolic disorder in pregnant patients [26]. Studies have shown that the incidence of gestational diabetes is increasing, which may result from postponing procreation plans for later years and the growing epidemic of obesity, diabetes and pre-diabetes states in the general population [27,28,29]. Variation in prevalence rates of GDM could be related to different diagnostic criteria used for screening and diversity of the populations being studied. The prevalence ranges from less than 2% in Sweden to 20.6% in United Arab Emirates [30]. GDM is a worldwide metabolic disorder that has negative maternal and neonatal effects with long-term consequences [31,32]. It is associated with adverse maternal health outcomes such as gestational hypertension, pre-eclampsia, caesarean section, and neonatal outcomes including hyperinsulinemia, macrosomia (usually defined as a neonate weighing over 4 kg), shoulder dystocia, and hypoglycemia. GDM is also a risk factor for future maternal obesity, type 2 diabetes and cardiovascular disease [33,34]. In most developed countries universal screening for GDM is preferred using fasting glucose and OGTT for diagnosis [35]. In a recent meta-analysis it was demonstrated that women with GDM had 7.43 times the likelihood of developing T2DM as pregnant women without GDM [7]. In normal pregnancy, insulin resistance increases in the second trimester, but most women remain euglycemic due to beta cell compensation and increased insulin secretion. GDM develops when beta cell compensation is inadequate for the hepatic glucose production and the level of insulin resistance [36]. Some authors have even demonstrated a reduction of pancreatic β-cell function by 67% in women with GDM compared with normal glucose tolerance controls [37].

### 4.1. CTRP-3

C1q/tumor necrosis factor related protein-3 (CTRP 3) is a novel adipokine belonging to the CTRP family and mainly secreted by mesenteric adipose tissue in humans [38]. Currently it is considered to be a crucial hormone involved in glucose and lipid metabolism [39]. It activates adenosine monophosphate-activated protein kinase (AMPK) and improves insulin signaling plus insulin sensitivity [12]. Furthermore, CTRP3 reduces secretion of inflammatory cytokines from 3T3-L1 adipocytes [40]. CTRP3 expression can decline in insulin resistance, where treatment with glucagon-like peptide-1 (GLP-1) receptor agonist enhances its expression and improves insulin sensitivity [41]. The association of circulating CTRP-3 with diabetes mellitus has been reported [39,42]. However, there are conflicting results considering concentration of CTRP-3 in diabetes mellitus. Qu et al. reported decreased circulating levels of CTRP-3 in patients with type 2 diabetes, while others showed increased levels of CTRP-3 in subjects with type 2 diabetes [42,43]. Ban et al. examined circulating CTRP-3 levels before and two hours after a glucose load and observed that in type 2 diabetic patients CTRP3 levels decreased from about 150 to 50 ng/ml in response to oral glucose load [39]. In this study glucose and insulin concentrations were significantly higher after the two hours OGTT, so both glucose and insulin could account for the reduction in serum CTRP-3 concentrations. Unfortunately, in the analyzed literature, we did not find any research on the concentration of CTRP-3 in GDM.

Choi et al. reported elevated CTRP-3 concentrations in prediabetes and type 2 diabetes compared with a normal glucose tolerance group [43]. In our study there was also a significantly higher concentration of CTRP-3 evaluated in the peripheral blood serum in patients with GDM compared to those in the control group. Higher concentration of CTRP-3 was observed in both subgroups (GDM1 and GDM2). Elevated values of this marker may suggest that these women are at increased risk of developing type 2 diabetes in a feature life. Similarity of the results of CTRP3 concentrations obtained in patients with GDM, prediabetic states and diabetes confirm that GDM is a real harbinger of future diabetes mellitus development. In a recent Chinese study, plasma CTRP-3 concentrations were significantly lower in subjects with pre-diabetes and type 2 diabetes mellitus compared with a normal glucose tolerance group [44]. A multiple linear regression analysis showed the plasma CTRP-3 levels were independently associated with homeostasis model assessment for insulin resistance (HOMA-IR). Further multiple logistical analyses indicated that plasma CTRP-3 concentrations were significantly correlated with prediabetes states and type 2 diabetes mellitus after adjusting for potential confounders. These results may indicate that CTRP-3 is an independent and strong predictor for prediabetes and diabetes [44].

Recent clinical studies demonstrate that the concentrations of CTRP-3 are lower in obese patients [45]. In the current study we also found weak, negative correlation between CTRP-3 concentration and current weight as well as increase of weight during pregnancy in women with GDM1. Interesting results were provided by the Wagner et al. study. They reported that CTRP-3 level is elevated in obese male but reduced in obese female subjects [46]. This gender specific regulation and function of CTRP3 requires further research. In the study by Moradi et al., CTRP3 demonstrated a negative correlation with HOMA-IR in type 2 diabetes cases [47]. It could be caused by the effect of insulin resistance on the expression of this adipokine in adipose tissue. Our research has not confirmed this relationship which may indicate the presence of other mechanisms regulating CTRP-3 levels during pregnancy.

### 4.2. PEDF

Pigment epithelium-derived factor (PEDF) is a 50-kDa glycoprotein belonging to the serine protease inhibitor (serpin) supergene family, located on chromosome 17p13.1 [48]. It is known as a pleiotropic protein which was first extracted from the medium of human fetal retinal pigment epithelium [49]. Mainly liver and adipose tissue are responsible for the production of circulating PEDF; however, it is expressed in most tissues examined [15]. Li et al. showed that serum PEDF, measured at 24–32 weeks of gestation, was elevated in pregnant women with GDM compared to those without GDM, which is probably an early detection marker for predicting development of GDM to type 2 diabetes mellitus [25]. Furthermore, univariate correlation data in pregnant women demonstrated that serum PEDF level was positively related with fasting glucose and HOMA-IR [25]. PEDF is not only associated with insulin sensitivity and diabetes mellitus but also with its complications [16,50,51].

PEDF has been found to be elevated in type 2 diabetes, polycystic ovarian syndrome and metabolic syndrome but the role of PEDF in diabetes is not well understood and needs to be further researched [52,53,54,55]. Some authors postulate that dysregulation of the PEDF-ATGL interaction may be associated with the elevation of PEDF level in serum. ATGL is crucial for lipid homeostasis lipase and putative PEDF receptor at the same time [56,57]. Our study did not reveal any statistically significant difference between the concentration of PEDF in the control and GDM groups as well as between subgroups with GDM1 and GDM2. The result of this study can be explained by the fact that PEDF concentration increases in more advanced stages of diabetes when typical diabetic complications develop, but not in GDM cases [50,51].

In the literature it is noted that insulin resistance and higher concentration of PEDF depend on obesity [52,58,59]. Nowadays, it is obvious that adipose tissue is not only a storage site for triglycerides but also an important endocrine organ [60].

Adipocytes release adipokines and contribute to a chronic low-grade inflammation state. PEDF expression in adipose tissue positively correlates with obesity and insulin resistance in mice. It is highly probable that the combination of obesity with hyperinsulinemia lead to increased PEDF serum concentration [61]. The results of human studies are consistent with the above mentioned report and describe a significant correlation between PEDF and obesity [55,62].

The mechanisms of how PEDF induces insulin resistance are not well understood. One of the factors contributing to insulin resistance is inflammation. PEDF is characterized by proinflammatory actions in several cell types [63]. In our study only a weak correlation between PEDF and a body weight (initial weight) was noted in GDM group. PEDF concentration was also weakly and negatively correlated with BMI (assessed before pregnancy). This may indicate that in GDM patients weight and weight gain do not influence PEDF concentrations as they do in chronic diabetes.

There were some limitations to this study. First, it was performed using only one sample of blood in the third trimester. Secondly, because this study included only European subjects, our results may not apply to other populations.

## 5. Conclusions

In conclusion, CTRP-3 concentrations were significantly higher in patients with GDM than the normal glucose tolerance group, whereas PEDF levels were not different. Due to the fact that CTRP-3 concentrations are elevated in GDM, further research is needed on the use of this parameter in the diagnosis of GDM.

## Figures and Tables

**Table 1:** Characteristic of the study and control groups. Comparison of the distribution of selected clinical factors according to groups.

Variable	Control (C)	GDM1	GDM2	GDM (1 + 2)	C vs. GDM (1 + 2)	C vs. GDM1	C vs. GDM 2	GDM1 vs. GDM2
	*n* = 54 (%)	*n* = 75 (%)	*n* = 43 (%)	*n* = 118 (%)	*p*			
**Pregnancy [n]**					0.4871	0.1312	0.3586	**0.0115**
1	26 (48.15)	49 (65.33)	16 (37.21)	65 (55.08)				
2	18 (33.33)	15 (20.00)	14 (32.56)	29 (24.58)				
>2	10 (18.52)	11 (14.67)	13 (30.23)	24 (20.34)				
**Labor [n]**					0.7852	0.2368	0.4006	**0.0138**
1	32 (59.26)	55 (73.33)	20 (46.51)	75 (63.56)				
2	15 (27.78)	13 (17.33)	14 (32.56)	27 (22.88)				
>2	7 (12.96)	7 (9.33)	9 (20.93)	16 (13.56)				
**Labor Type**					0.5117	0.4311	0.8931	0.7283
Vaginal delivery	22 (40.74)	37 (49.33)	19 (44.19)	56 (47.46)				
Caesarean section	32 (59.26)	38 (50.67)	24 (55.81)	62 (52.54)				
**Preterm Labor**					0.1086	0.3913	**0.0282**	0.1782
No	53 (98.15)	70 (93.33)	36 (83.72)	106 (89.83)				
Yes	1 (1.85)	5 (6.67)	7 (16.28)	12 (10.17)				
**Sex of the Newborn**					0.6354	0.9901	0.1428	0.0815
Girl	28 (51.85)	40 (53.33)	15 (34.88)	55 (46.61)				
Boy	26 (48.15)	35 (46.67)	28 (65.12)	63 (53.39)				
**APGAR 1**					0.0125	**0.0006**	0.6296	**0.0279**
10	36 (66.67)	26 (33.77)	25 (58.14)	51 (43.22)				
9	15 (27.78)	35 (45.45)	14 (32.56)	49 (41.53)				
<9	3 (5.55)	16 (20.78)	4 (9.30)	18 (15.25)				
**BMI (before Pregnancy) [kg/m ^2^** **]**					**0.0014**	**0.0055**	**0.0005**	0.1287
<18.50	3 (5.56)	6 (8.00)	-	6 (5.08)				
18.50–24.99	45 (83.33)	43 (57.33)	24 (55.81)	67 (56.78)				
>24.99	6 (11.11)	26 (34.67)	19 (44.19)	45 (38.14)				

C-control group, GDM-gestational diabetes mellitus, BMI-body mass index.

**Table 2:** Comparison of values of selected factors between different groups.

Variable	Control (C) (*n* = 54)	GDM1 (*n* = 75)	GDM2 (*n* = 43)	GDM (1 + 2) (*n* = 118)	C vs. GDM (1 + 2)	C vs. GDM1 vs. GDM2
	*Me* (Interquartile Range)				*p*	
**Age [years]**	**29.00 (26.00–34.00)**	**32.00 (28.00–35.00)**	**31.00 (27.00–36.00)**	32.00 (28.00–35.00)	0.0733	0.1980
**Glucose [mg/dL]**	60.04 (52.72–66.21)	67.11 (51.95–75.21)	66.43 (55.98–76.75)	66.62 (54.70–75.92)	**0.0013**	**0.0055** (C vs. GDM1; C vs. GDM2)
**Insulin [uLU/mL]**	11.77 (7.44–17.98)	11.40 (6.69–18.44)	12.94 (8.43–22.68)	12.50 (8.18–19.67)	0.6776	0.3761
**OGTT (Fasting) [mg/dL]**	79.50 (77.00–84.00)	82.00 (79.00–88.00)	88.00 (82.00–93.00)	85.00 (80.00–89.00)	**<0.0001**	**<0.0001** (C vs. GDM1; C vs. GDM2; GDM1 vs. GDM2)
**OGTT (75g 2 h) [mg/dL]**	110.00 (93.00–119.00)	158.00 (155.00–162.00)	160.00 (155.00–169.00)	159.00 (155.00–165.50)	**<0.0001**	**<0.0001** (C vs. GDM1; C vs. GDM2)
**HOMA-IR**	1.58 (1.22–2.50)	1.75 (1.15–3.11)	2.05 (1.48–3.67)	1.96 (1.18–3.43)	0.0954	0.0857
**PEDF [ng/mL]**	40.67 (31.05–119.37)	38.25 (32.54–163.24)	54.96 (28.91–186.57)	42.07 (32.25–163.24)	0.9789	0.8543
**CTRP3 [ng/mL]**	4.79 (2.74–12.12)	8.40 (1.99–18.66)	10.96 (3.17–25.59)	8.84 (2.60–18.98)	**0.0265**	**0.0178** (C vs. GDM1; C vs. GDM2)
**Pregnancy [*n*]**	2.00 (1.00–2.00)	1.00 (1.00–2.00)	2.00 (1.00–3.00)	1.00 (1.00–2.00)	0.8070	**0.0421** (GDM1 vs. GDM2)
**Labor [*n*]**	1.00 (1.00–2.00)	1.00 (1.00–2.00)	1.50 (1.00–2.00)	1.00 (1.00–2.00)	0.6349	0.1116
**Gestational Age at Birth [weeks]**	40.00 (38.60–41.00)	39.00 (38.00–39.00)	38.00 (37.00–39.00)	38.40 (37.55–39.00)	**<0.0001**	**<0.0001**
**Birth weight of the Newborn [g]**	3435.00 (3170.00–3840.00)	3370.00 (3100.00–3790.00)	3400.00 (3130.00–3650.00)	3375.00 (3100.00–3780.00)	0.3698	0.6619
**APGAR 1**	10.00 (9.00–10.00)	9.00 (9.00–10.00)	10.00 (9.00–10.00)	9.00 (9.00–10.00)	0.0590	**0.0029** (C vs. GDM1; GDM1 vs. GDM2)
**APGAR 5**	-	-	10.00 (5.00–10.00)	10.00 (5.00–10.00)	1.0000	1.0000
**BMI (before pregnancy) [kg/m^2^]**	23.28 (21.48–24.38)	23.23 (21.48–25.78)	24.22 (22.77–27.82)	23.44 (22.09–26.15)	**0.0341**	**0.0164** (C vs. GDM2; GDM1 vs GDM2)
**Weight (Baseline) [kg]**	65.00 (59.00–69.00)	65.00 (60.00–75.00)	66.00 (60.00–76.00)	65.00 (60.00–75.00)	0.0566	0.1431
**Weight (Current) [kg]**	76.50 (72.00–83.00)	79.50 (74.00–85.00)	78.50 (72.50–86.50)	79.00 (74.00–86.00)	0.1048	0.2640
**Height [cm]**	167.00 (163.00–171.00)	167.00 (164.00–170.00)	166.50 (162.00–170.00)	167.00 (164.00–170.00)	0.9945	0.7559
**Increase of Weight during Pregnancy [kg]**	14.00 (11.00–16.00)	13.00 (12.00–15.00)	11.50 (7.00–14.00)	12.50 (10.00–15.00)	0.1066	0.1213

Data were presented as the median (*Me*) and interquartile range. C-control group, GDM-gestational diabetes mellitus, BMI-body mass index, OGTT-oral glucose tolerance test, PEDF-pigment epithelium-derived factor, CTRP-3C1q/tumor necrosis factor-related protein-3.

**Table 3:** Correlation between selected factors and Pigment Epithelium-Derived Factor (PEDF) [ng/mL].

Variable	Control		GDM1		GDM2		GDM (1 + 2)	
	rho	*p*	rho	*p*	rho	*p*	rho	*p*
**Age [years]**	−0.412	**0.0020**	−0.074	0.5420	−0.103	0.5119	−0.097	0.3053
**Glucose [mg/dL]**	0.012	0.9306	0.024	0.8377	−0.142	0.3648	−0.038	0.6860
**Insulin [uLU/mL]**	0.008	0.9553	−0.089	0.4470	0.110	0.4820	0.008	0.9287
**OGTT (Fasting) [mg/dL]**	0.041	0.7691	0.059	0.6149	0.087	0.5817	0.046	0.6267
**OGTT (75 g 2 h) [mg/dL]**	−0.086	0.5363	0.114	0.3342	0.239	0.1268	0.156	0.0940
**HOMA-IR**	−0.006	0.9657	−0.061	0.6040	0.038	0.8067	−0.001	0.9878
**Pregnancy [n]**	−0.205	0.1407	−0.128	0.2933	0.061	0.7026	−0.033	0.7283
**Labor [n]**	−0.220	0.1129	−0.104	0.3967	−0.001	0.9937	−0.053	0.5857
**Gestational Age [weeks]**	0.035	0.8048	−0.045	0.7089	0.001	0.9967	−0.057	0.5525
**Birth weight of the Newborn [g]**	0.066	0.6402	0.095	0.4356	0.042	0.7949	0.012	0.9040
**APGAR 1**	−0.146	0.3343	0.059	0.6646	0.330	0.0699	0.172	0.1098
**APGAR 2**	-	-	-	-	0.825	**0.0117**	0.825	**0.0117**
**BMI (before Pregnancy) [kg/m^2^]**	−0.079	0.5693	−0.019	0.8748	−0.351	**0.0210**	−0.128	0.1711
**Weight (Initial) [kg]**	0.001	0.9961	−0.295	0.0719	−0.191	0.2381	−0.229	**0.0438**
**Weight (Current) [kg]**	0.075	0.5920	−0.019	0.8761	−0.143	0.3799	−0.101	0.2930
**Height [cm]**	0.226	0.0999	0.114	0.3441	0.052	0.7504	0.056	0.5601
**Increase of Weight during Pregnancy [kg]**	0.290	0.0504	−0.313	0.0556	0.095	0.5600	−0.083	0.4696

**Table 4:** Correlation between selected factors and CTRP3 [ng/ml].

Variable	Control		GDM1		GDM2		GDM (1 + 2)	
	rho	*p*	rho	*p*	rho	*p*	rho	*p*
**Age [years]**	−0.081	0.5631	0.117	0.3382	−0.014	0.9265	0.043	0.6499
**Glucose [mg/dL]**	0.043	0.7593	−0.020	0.8654	−0.087	0.5812	−0.048	0.6037
**Insulin [uLU/mL]**	−0.215	0.1225	0.145	0.2164	0.333	**0.0293**	0.208	**0.0243**
**OGTT (Fasting) [mg/dl]**	−0.096	0.4953	0.095	0.4249	0.136	0.3920	0.129	0.1706
**OGTT (75 g 2 h) [mg/dL]**	−0.145	0.3003	−0.136	0.2517	0.029	0.8567	−0.034	0.7155
**HOMA-IR**	−0.221	0.1125	0.124	0.2943	0.240	0.1214	0.171	0.0651
**PEDF [ng/mL]**	0.111	0.4300	0.210	0.0731	0.252	0.1026	0.158	0.0892
**Pregnancy [n]**	−0.060	0.6729	−0.001	0.9951	0.062	0.6954	0.019	0.8417
**Labor [n]**	−0.088	0.5336	0.053	0.6671	0.099	0.5449	0.077	0.4277
**Gestational Age [weeks]**	0.005	0.9742	−0.163	0.1815	−0.382	**0.0126**	−0.254	**0.0070**
**Birth Weight of the Newborn [g]**	−0.045	0.7531	−0.068	0.5809	0.000	0.9989	−0.050	0.6090
**APGAR 1**	0.115	0.4510	−0.214	0.1136	0.100	0.5935	−0.052	0.6356
**APGAR 2**	-	-	-	-	0.536	0.1708	0.536	0.1708
**BMI (before Pregnancy) [kg/m^2^]**	0.075	0.5926	−0.113	0.3433	−0.037	0.8123	−0.073	0.4389
**Weight (Baseline) [kg]**	−0.132	0.3819	−0.211	0.2096	0.121	0.4568	−0.011	0.9252
**Weight (Current) [kg]**	0.008	0.9542	−0.256	**0.0341**	0.188	0.2465	−0.080	0.4081
**Height [cm]**	−0.086	0.5423	−0.173	0.1512	0.236	0.1433	−0.033	0.7327
**Increase of Weight during Pregnancy [kg]**	0.027	0.8610	−0.352	**0.0326**	0.126	0.4381	−0.052	0.6507
